# A survey of factors influencing the intention to undergo genetic testing in patients with cardiovascular disease in Japan

**DOI:** 10.1002/jgc4.70213

**Published:** 2026-04-24

**Authors:** Tomomi Miyoshi, Atsushi Mizuno, Masaki Watanabe, Koichiro Isa, Kanna Fujita, Toru Kubo, Seitaro Nomura

**Affiliations:** ^1^ Division of Public Health, Department of Social Medicine Nihon University School of Medicine Tokyo Japan; ^2^ Department of Cardiovascular Medicine St. Luke's International Hospital Tokyo Japan; ^3^ Graduate School of Teacher Education Tokyo Gakugei University Tokyo Japan; ^4^ Department of Frontier Cardiovascular Science, Graduate School of Medicine The University of Tokyo Tokyo Japan; ^5^ Department of Cardiology and Geriatrics, Kochi Medical School Kochi University Kochi Japan; ^6^ Department of Cardiovascular Medicine, Graduate School of Medicine The University of Tokyo Tokyo Japan

**Keywords:** behavioral intention, cardiovascular disease, genetic testing, genomic literacy, health numeracy, patient decision‐making

## Abstract

Although genetic testing for cardiovascular disease (CVD) can enhance precision medicine and optimize treatment decisions, its clinical application remains limited. This study aimed to examine the associations of genomic knowledge (GK), health numeracy (HN), and perceptions of genetic testing (PGT) with behavioral intentions toward genetic testing among individuals with self‐reported physician‐diagnosed CVD. An online cross‐sectional survey of 840 Japanese individuals with self‐reported physician‐diagnosed CVD was conducted in October 2023. We analyzed the relationships among GK, HN, PGT, and two behavioral intentions (i.e., undergoing testing and sharing results with family). Responses from 748 individuals with CVD (65.6% men; mean age = 57.5 years, SD = 15.8; 41.0% with a family history of CVD) were analyzed. Multiple regression analysis indicated that age, family history of CVD, and perceived usefulness were associated with the behavioral intentions toward genetic testing. The structural equation modeling (SEM) path model suggested that GK and HN were indirectly related to intention via perceived usefulness. In the SEM path model, perceived usefulness showed strong positive associations with both testing intention (β = 0.70, *p* < 0.001) and sharing intention (β = 0.63, *p* < 0.001). Fear was negatively associated with testing intention (β = −0.14, *p* < 0.001), whereas its association with sharing intention was small (β = −0.06) and not robust in bootstrapping. Behavioral intentions regarding genetic testing for CVD were more strongly associated with perceived usefulness, and a family history of CVD may be associated with differences in perceptions and intentions, potentially through knowledge‐related pathways. As the associations were correlational, the findings highlight the need for counseling approaches that integrate the cognitive and emotional aspects of genomic literacy.

**What is known about this topic**

Genetic testing for inherited cardiovascular conditions can inform diagnosis, risk stratification, and cascade testing; however, its uptake remains limited. Decision‐making regarding genetic testing has been linked to cognitive factors (e.g., knowledge and numeracy) and perceptions (e.g., perceived benefits and emotional responses); however, evidence among individuals with established cardiovascular disease is limited.

**What this paper adds to the topic**

In a web‐based survey of Japanese individuals with self‐reported cardiovascular disease recruited via a national online panel, perceived usefulness showed the strongest association with both the intention to undergo genetic testing and to share results with family. Genomic knowledge and health numeracy were associated with both intentions, primarily indirectly through perceived usefulness. Family history was associated with higher knowledge, greater perceived usefulness, and stronger intentions, whereas fear was negatively associated with testing intention.

## 1 INTRODUCTION

Genetic testing for cardiovascular disease (CVD) has transformed clinical practice by improving diagnostic accuracy, identifying heritable mutations, and supporting risk stratification and targeted therapies. Recent international society guidance—including the 2022 EHRA/HRS/APHRS/LAHRS expert consensus statement and the 2023 European Society of Cardiology (ESC) guidelines for cardiomyopathies—recommends cardiogenetic testing (typically multi‐gene panel testing), particularly when an inherited cardiomyopathy or inherited arrhythmia syndrome is suspected (Arbelo et al., 2023; Wilde et al., 2022).

The diagnostic yield varies substantially by clinical indication, phenotype, and family history and can be substantial in inherited cardiomyopathies and inherited arrhythmia syndromes. Representative yields reported in the literature range from approximately one‐fifth to one‐half in dilated cardiomyopathy and can be higher in selected inherited arrhythmia syndromes (Wilde et al., 2022). However, its clinical use remains limited. In a real‐world analysis, genetic testing was documented in only a small proportion of patients with dilated and hypertrophic cardiomyopathy, indicating that uptake remains limited in routine care (Longoni et al., 2023). In Japan, contemporary reports similarly describe the underutilization and structural barriers to implementation in routine practice (Isa et al., 2024; Kubo & Kitaoka, 2023). This limited utilization suggests that factors beyond clinical eligibility, such as patient knowledge, numeracy, and perceptions, may play important roles in the decision‐making process.

Cardiogenetic testing shares important similarities with hereditary cancer genetic testing, as the results can inform individual risk management and enable preventive measures for relatives through cascade testing (Schmidlen et al., 2022). Simultaneously, counseling on inherited cardiac conditions may require additional considerations—such as variable and age‐dependent penetrance and implications for the longitudinal surveillance of at‐risk relatives (Arbelo et al., 2023; Wilde et al., 2022). Consequently, patients' understanding of the implications for themselves and their families can shape their willingness to undergo testing and share their results. Prior studies suggest that genetic testing decisions are influenced by both cognitive factors (e.g., knowledge and perceived benefits) and affective factors (e.g., perceived risk and emotional responses such as fear, worry, or anxiety) (DiLorenzo et al., 2006; Oliveri et al., 2018; Sweeny et al., 2014).

In this study, “cardiogenetic testing” primarily refers to multi‐gene panel testing used in the evaluation of suspected inherited cardiomyopathies and inherited arrhythmia syndromes. In this context, its clinical utility includes etiologic clarification, risk stratification/management, and cascade testing for relatives (Arbelo et al., 2023; Cirino et al., 2017; Wilde et al., 2022). Recent reports from Japan suggest that the implementation of cardiogenetic testing in routine practice remains limited and faces structural challenges (Isa et al., 2024; Kubo & Kitaoka, 2023). In addition, the 2024 Japanese guideline provides an updated framework for genetic testing and counseling in CVD (Imai et al., 2024).

In recent years, the concept of genomic literacy has been proposed to describe the ability to understand and use genetic information in health‐related decisions. Miyoshi and Watanabe conceptualized genomic literacy as a multidimensional construct that includes genetic/genomic knowledge (GK), health numeracy (HN), and interactive and critical health literacy (Miyoshi & Watanabe, 2023). In the present study, we focused on GK and HN as core cognitive components relevant to cardiogenetic testing decisions. GK refers to a factual understanding of genetic principles, such as inheritance, risk, and the probabilistic nature of test results, whereas HN refers to the ability to comprehend and apply numerical and probabilistic information relevant to health contexts (Miyoshi & Watanabe, 2022; Peters, 2008). Together, these competencies form a foundation for interpreting cardiogenetic test information and considering its implications for oneself and one's family members.

However, knowledge alone may not be sufficient for informed decision‐making. Behavioral science frameworks, such as the Health Belief Model (HBM) and Protection Motivation Theory (PMT), posit that perceived benefits and threats (including perceived risk and fear) shape health‐related intentions and behaviors (Janz & Becker, 1984; Rogers, 1975; Rosenstock, 1974). Risk perceptions are known to influence health behaviors through both deliberative and affective pathways (Ferrer & Klein, 2015). In the context of genetic testing, perceptions of genetic testing (PGT)—including perceived usefulness and fear—may function as proximal determinants of intentions, potentially mediating the association between genomic literacy and behavioral outcomes (Helmes, 2002; Oliveri et al., 2018; Sweeny et al., 2014).

Although previous studies have examined the predictors of public attitudes toward genetic testing, relatively few have focused on individuals already diagnosed with CVD, whose decision‐making context may involve greater personal and familial relevance. Research on decision‐making related to cardiogenetic testing in CVD populations remains limited. In Japan, available reports have mainly described implementation issues and demographic correlates of testing uptake rather than the cognitive and perceptual pathways underlying patients' intentions (Isa et al., 2024; Kubo & Kitaoka, 2023). Moreover, while associations between genomic literacy and general genetic testing attitudes have been reported, the mediating role of perceptions—particularly perceived usefulness and fear—has not been well tested in cardiogenetic contexts (Miyoshi & Watanabe, 2023; Sweeny et al., 2014).

Furthermore, because genetic results may reveal heritable risks affecting multiple relatives, individuals may experience both motivation (e.g., to protect family members) and apprehension (e.g., stigma, discrimination, or anxiety). Concerns about genetic discrimination and its impact on testing‐related decisions have been documented in genetic testing contexts (Barlow‐Stewart et al., 2009; Green & Botkin, 2003). Therefore, a family history of CVD may be an important contextual factor related to knowledge, perceived relevance, and perceptions regarding testing (Davison et al., 1992; Hunt et al., 2000).

Building on these theoretical and empirical foundations, this study focuses on three interrelated components—GK, HN, and PGT—as determinants of intention to undergo cardiogenetic testing. We hypothesized that higher levels of GK and HN would be associated with more favorable perceptions of the usefulness of genetic testing, and that perceived usefulness would be positively associated, whereas fear would be negatively associated with intentions to undergo testing and share results with the family. We also examined family history of CVD and age as antecedent variables influencing knowledge and perceptions, using a correlational path model to estimate direct and indirect pathways without causal interpretation.

This study aimed to identify the factors associated with the intention to undergo and share the results of cardiogenetic testing among individuals with CVD in Japan. By integrating genomic literacy and perception‐based constructs into a single path model, this study extends prior work by focusing on a clinical population and clarifying how cognitive capacities and perceptions jointly relate to testing intentions, with particular attention to family history.

## 2 METHODS

### 2.1 Study design and participants

This cross‐sectional, anonymous, self‐administered online survey was conducted in October 2023 among Japanese residents aged ≥15 years who self‐reported a physician diagnosis of CVD. Participants were recruited from the panel of a web‐based survey company, and eligibility was confirmed using a screening question at the start of the survey, asking whether they had ever been diagnosed by a physician with any of the listed CVD conditions. Medical records were not available for verification. Eligible conditions included heart failure, cardiomyopathy, angina pectoris, myocardial infarction, arrhythmia, valvular heart disease, congenital heart disease, transient ischemic attack, cerebral infarction, cerebral hemorrhage, subarachnoid hemorrhage, peripheral vascular disease, pulmonary thromboembolism, aortic disease, pulmonary hypertension, and hypertension (Table S1). All respondents included in the analytic sample reported at least one eligible CVD diagnosis other than hypertension (i.e., none were included based on hypertension).

An inclusive definition of CVD was adopted to reflect the diversity of cardiovascular conditions encountered in clinical practice and to capture a wide range of experiences relevant to genetic testing decisions. Although the genetic contribution varies across these diseases, cardiogenetic testing decisions are increasingly being discussed in various cardiovascular contexts, including multifactorial or acquired conditions. To assess robustness with respect to genetic relevance, we conducted sensitivity analyses stratified by cardiovascular condition group (Table S2), which showed broadly consistent patterns of association.

Participants were recruited through a web‐based research company that maintained a large national panel of registered users. Participants who completed the survey received a modest incentive (redeemable points) provided by the survey company in accordance with its standard panel policy; the researchers did not provide any additional incentives. Demographic quotas for sex and prefectural distribution were applied based on national statistics to mitigate the sampling bias. These quotas were intended to balance participant attributes rather than ensure full population representativeness. Because the prevalence of CVD increases with age, sampling was stratified by age group to capture diverse disease experiences rather than to reflect the population prevalence. Because participation required panel registration and internet access, respondents may differ from clinic‐based patient populations (e.g., in disease severity, access to specialty care, and health/digital literacy); therefore, the generalizability of absolute levels of knowledge and intentions should be interpreted with caution. Of the 840 invited eligible panelists, 748 completed the questionnaire (response rate, 89.0%) and provided valid data for analysis.

Participants provided electronic informed consent prior to completing the questionnaire. No standardized definition or explanatory description of cardiogenetic testing was provided prior to the relevant survey items; participants responded based on the item stems only.

### 2.2 Questionnaire development

The questionnaire was developed to assess five domains: (1) demographic and clinical attributes, (2) GK, (3) HN, (4) PGT, and (5) behavioral intentions related to genetic testing for CVD. Items were reviewed by six experts to assess clarity and content relevance and were subsequently pretested among lay participants (*n* = 12), and minor wording revisions were made.

#### 2.2.1 Demographic and clinical attributes

Respondents reported their sex, age, marital status, presence of children, occupation, highest educational attainment, healthcare profession involvement, and family history of CVD (assessed based on self‐report of whether any first‐ or second‐degree relative had been diagnosed with CVD).

#### 2.2.2 Genomic knowledge (GK)

GK was assessed using 10 author‐developed dichotomous items designed to capture core genetic concepts relevant to health decisions (e.g., inheritance and interpretation of genetic information) and tailored to CVD contexts, with reference to previous studies on genomic literacy in Japan (Miyoshi & Watanabe, 2021a, 2023). Each item was scored as 1 (“correct”) or 0 (“incorrect/don't know”), and the scores were summed to yield a total GK score ranging from 0 to 10. Internal consistency was evaluated using the Kuder–Richardson Formula 20 (KR‐20), which was acceptable (KR‐20 = 0.82; 10 items; *N* = 748).

#### 2.2.3 Health numeracy (HN)

HN was assessed using six author‐developed dichotomous objective items on proportions, percentages, and probabilities in health contexts, developed with reference to prior work on HN in Japan (Miyoshi & Watanabe, 2022), the Numeracy Understanding in Medicine Instrument (NUMi) (Schapira et al., 2012), and numeracy‐related risk communication research (Peters, 2008). Responses were scored as 1 (“correct”) or 0 (“incorrect/don't know”) and summed to yield total scores ranging from 0 to 6. Internal consistency was evaluated using the Kuder–Richardson Formula 20 (KR‐20) and was acceptable (KR‐20 = 0.78; 6 items; *N* = 748).

#### 2.2.4 Perceptions of genetic testing (PGT)

PGT were measured using four items developed with reference to the Japanese national guidance on genetic testing and diagnosis in medical practice (The Japanese Association of Medical Sciences, 2022). Perceived usefulness was assessed with three items: (Usefulness A) “Genetic testing can reduce the risk of any disease”; (Usefulness B) “Knowing the results of genetic testing is useful for my future, such as planning my life”; and (Usefulness C) “Genetic testing may reveal abnormalities (i.e., clinically meaningful genetic findings/variants).” Perceived fear was assessed with a single item: “I feel scared about taking a genetic test.” Responses were rated on a 5‐point Likert scale (1 = strongly disagree, 5 = strongly agree). The three‐item usefulness scale showed modest internal consistency (Cronbach's α = 0.65; 3 items; *N* = 748) and was summed to yield a total score ranging from 3 to 15, with higher scores indicating greater perceived usefulness. The fear item ranged from 1 to 5, with higher scores indicating greater perceived fear. Internal consistency was not assessed for this single‐item measure. As noted above, no standardized definition or explanatory description of cardiogenetic testing was provided prior to these items; participants responded based on the item wording referring to “genetic testing for cardiovascular disease (CVD).”

#### 2.2.5 Behavioral intentions toward genetic testing

Behavioral intentions were assessed using two items: (Intention A; testing intention) “Would you be willing to undergo genetic testing for cardiovascular disease (CVD)?” and (Intention B; sharing intention) “Would you be willing to share the results of genetic testing for CVD with your family?” Each item was rated on a 5‐point Likert scale (1 = not at all, 5 = very much so). The two items were analyzed separately to capture distinct behavioral dimensions.

No standardized definition or explanatory description of cardiogenetic testing was provided prior to these items; participants responded based on the item stems only.

### 2.3 Statistical analyses

All statistical analyses were conducted using IBM SPSS Statistics (version 29.0) and AMOS (version 29.0) (IBM Corp., Armonk, NY, USA). Descriptive statistics (mean, standard deviation, and proportion) were calculated for all study variables.

Group differences in GK, HN, PGT, and behavioral intentions were examined using independent‐sample *t*‐tests (for dichotomous variables) or one‐way analysis of variance (ANOVA) for multi‐category variables. Effect sizes were reported as Cohen's *d* and *η* ^2^, respectively.

Pearson's product–moment correlations were computed to assess associations among continuous variables. Two multiple linear regression analyses were performed to examine factors associated with behavioral intentions. As sensitivity analyses, we conducted stratified regression models by cardiovascular condition group using parsimonious models that focused on the core constructs (Table S2). Specifically, we modeled (i) perceived usefulness (summed Usefulness A–C) and perceived fear as outcomes predicted by GK and HN, and (ii) Intention A and Intention B as outcomes predicted by GK, HN, perceived usefulness (summed), and perceived fear. These stratified models were used to assess the consistency of key associations across strata. In the main multiple regression models, we entered the three usefulness items (Usefulness A–C) separately to examine item‐specific associations, whereas in sensitivity analyses, we used the summed usefulness score (A–C; range 3–15) to reduce model complexity; in the structural equation modeling (SEM) path model, perceived usefulness was modeled as a latent construct indicated by the three items. In sensitivity analyses, a proxy‐defined “predominant monogenic component” subgroup was defined as participants reporting heart failure and/or cardiomyopathy and/or arrhythmia, and an “acquired/multifactorial only” subgroup was defined as those reporting none of these conditions. We reported the standardized regression coefficients (β) with *p*‐values for each stratum. Model 1: Testing intention (dependent variable); Model 2: Sharing intention (dependent variable). In the main multiple regression analyses, we simultaneously entered sex, age group, marital status, parental status, education, healthcare‐related occupation, family history of CVD, GK, HN, the three usefulness items (Usefulness A–C), and fear. Variance inflation factors (VIFs) were examined to confirm the absence of multicollinearity (VIF < 2.0 for all variables). Standardized β coefficients and *p*‐values were reported, and the direction of each effect (positive or negative) is described in the “Results” section. To explore potential mediating relationships among cognitive and perceptual factors, an SEM path model was conducted based on the hypothesized model derived from the HBM and PMT frameworks. In this structural model, GK and HN were specified as cognitive antecedents, perceived usefulness and fear as mediators, and behavioral intentions as outcome variables. Model fit was evaluated using multiple indices (*χ* ^2^, GFI, AGFI, CFI, and RMSEA), considering conventional guidelines as references rather than strict cutoffs. Indirect effects were tested using bias‐corrected bootstrapping (5000 resamples). All analyses were two‐tailed with a significance level of *p* < 0.05. In addition, we inspected bias‐corrected bootstrap‐standardized 95% confidence intervals for key direct paths as a robustness check. The asterisks in the SEM path model figures indicate the ML‐based *p*‐values.

### 2.4 Ethical considerations

The study protocol was approved by the Research Ethics Committee of the Faculty of Medicine, The University of Tokyo (approval no. 2022166G). The web research company conducting the survey holds the PrivacyMark certification (Japan Institute for Promotion of Digital Economy and Community) and complies with ISO/IEC 27001 standards for information security. Only anonymized data were provided to the researchers. Respondents were informed that their participation was voluntary, that they could withdraw at any time, and that their data would be analyzed in aggregate form. As no clinical interventions were performed, the risk to the participants was minimal.

## 3 RESULTS

### 3.1 Participant characteristics

Of the 840 invited eligible respondents, 748 completed the questionnaire (response rate: 89.0%) and provided valid responses for analysis. The mean age was 57.5 years (SD = 15.8; range = 15–89 years). Men comprised 65.6% (*n* = 491) and women 34.4% (*n* = 257). A family history of CVD was reported in 41.0% (*n* = 307). The participant characteristics and group comparisons are summarized in Tables 1 and 2.

**TABLE 1:** Comparison of genomic knowledge and health numeracy scores by attributes.

	*n*	%	*N* = 748							
			Genomic knowledge score		*t*/*F*	*p* (effect size)	Health numeracy score		*t*/*F*	*p* (effect size)
			Mean	SD			Mean	SD		
Overall	748	100	4.67	2.95	—	—	3.95	1.72	—	—
Sex*										
Male	491	65.6	4.69	3.02	0.16	0.872 (0.012)	4.02	1.72	1.45	0.148 (0.112)
Female	257	34.4	4.65	2.82			3.82	1.72		
Age group (years)^a^										
Under 20	10	1.3	3.30	3.09	3.10^b^	0.008 (0.027)	2.80	1.75	13.77^b^	<0.001 (0.129)
20s	53	7.1	4.23	3.29			2.36	1.84		
30s	69	9.2	4.04	3.08			2.90	2.09		
40s	71	9.5	3.90	3.16			3.90	1.65		
50s	83	11.1	4.66	2.96			4.05	1.72		
60s	295	39.4	5.18	2.85			4.33	1.48		
Over 70	167	22.3	4.60	2.73			4.26	1.44		
Marital status*										
Married	516	69.0	4.73	2.94	0.76	0.449 (0.060)	4.10	1.66	3.49	<0.001 (0.284)
Unmarried	232	31.0	4.55	2.98			3.62	1.80		
Children*										
Yes	495	66.2	4.81	2.89	1.74	0.083 (0.137)	4.18	1.56	4.87	<0.001 (0.401)
No	253	33.8	4.41	3.06			3.50	1.91		
Level of education^a^										
Junior high school	26	3.5	2.62	2.83	5.37^b^	<0.001 (0.028)	2.88	2.08	2.55^b^	0.042 (0.018)
High school	204	27.3	4.30	2.76			3.86	1.69		
Technical school, technical college or junior college	127	17.0	5.06	3.02			3.98	1.55		
College	333	44.5	4.82	2.92			4.10	1.68		
Graduate school	58	7.8	5.21	3.25			3.81	2.02		
Occupation*										
Health care provider	13	1.7	7.92	2.25	5.21	<0.001 (1.132)	4.54	1.13	1.25	0.213 (0.349)
Non‐health care provider	735	98.3	4.62	2.93			3.94	1.72		
Family history of cardiovascular diseases*										
Yes	307	41.0	5.22	2.81	4.33	<0.001 (0.318)	4.16	1.55	2.91	0.004 (0.211)
No or unknown	441	59.0	4.29	2.99			3.80	1.81		

*Note*: Unpaired *t*‐test. Effect size: Cohen's *d* for *t*‐tests; *η* ^2^ for one‐way ANOVA.

Abbreviation: SD, standard deviation.

* Unpaired *t*‐test.

^a^ One‐way analysis of variance (ANOVA); Welch's ANOVA was used when the homogeneity of variance assumption was violated.

^b^ *F* value (Welch's *F* is reported for Welch's ANOVA).

**TABLE 2:** Comparison of perceived usefulness, perceived fear, and behavioral intentions by attributes.

	*n*	%	*N* = 748																							
			Perceived usefulness A		*t*/*F*	*p* (effect size)	Perceived usefulness B		*t*/*F*	*p* (effect size)	Perceived usefulness C		*t*/*F*	*p* (effect size)	Perceived fear		*t*/*F*	*p* (effect size)	Intention A		*t*/*F*	*p* (effect size)	Intention B		*t*/*F*	*p* (effect size)
			Mean	SD			Mean	SD			Mean	SD			Mean	SD			Mean	SD			Mean	SD		
Overall	748	100	3.08	1.06	—	—	3.48	0.93	—	—	3.16	1.07	—	—	3.41	1.01	—	—	3.11	1.13	—	—	3.33	1.12	—	—
Sex*																										
Male	491	65.6	3.09	1.09	0.36	0.720 (0.028)	3.44	0.95	−1.49	0.137 (0.115)	3.15	1.07	−0.53	0.594 (0.041)	3.37	0.97	−1.14	0.255 (0.088)	3.09	1.15	−0.83	0.408 (0.064)	3.31	1.14	−0.61	0.545 (0.047)
Female	257	34.4	3.06	1.01			3.55	0.89			3.19	1.08			3.46	1.08			3.16	1.08			3.37	1.08		
Age group (years)^a^																										
Under 20	10	1.3	3.10	1.29	2.91^b^	0.012 (0.022)	3.60	1.07	0.66^b^	0.682 (0.006)	2.90	1.52	4.53^b^	<0.001 (0.034)	3.80	1.14	1.71^b^	0.127 (0.016)	3.10	1.10	3.67^b^	0.002 (0.030)	3.20	1.55	1.42^b^	0.214 (0.001)
20s	53	7.1	3.28	1.32			3.53	1.08			3.36	1.42			3.62	1.21			3.45	1.08			3.57	1.14		
30s	69	9.2	3.33	1.00			3.49	0.99			3.45	1.09			3.57	1.16			3.32	1.10			3.38	1.13		
40s	71	9.5	3.37	0.91			3.62	0.90			3.48	1.04			3.58	1.09			3.48	1.11			3.59	1.06		
50s	83	11.1	2.94	1.07			3.46	0.89			3.24	1.01			3.46	0.93			3.17	1.07			3.29	1.14		
60s	295	39.4	3.05	1.02			3.49	0.87			3.13	1.05			3.32	0.94			3.01	1.10			3.30	1.06		
Over 70	167	22.3	2.92	1.08			3.37	0.99			2.89	0.92			3.29	0.95			2.92	1.18			3.21	1.18		
Marital status*																										
Married	516	69.0	3.08	1.07	−0.08	0.936 (0.006)	3.48	0.91	0.09	0.930 (0.007)	3.15	1.07	−0.48	0.634 (0.038)	3.40	1.00	−0.08	0.936 (0.006)	3.06	1.12	−1.82	0.069 (0.144)	3.37	1.08	1.55	0.121 (0.123)
Unmarried	232	31.0	3.09	1.05			3.47	0.98			3.19	1.07			3.41	1.02			3.22	1.14			3.24	1.20		
Children*																										
Yes	495	66.2	3.12	1.07	1.43	0.154 (0.110)	3.52	0.91	1.58	0.113 (0.122)	3.13	1.08	−1.09	0.278 (0.084)	3.40	1.02	−0.04	0.969 (0.003)	3.08	1.14	−1.00	0.318 (0.077)	3.38	1.12	1.51	0.130 (0.117)
No	253	33.8	3.00	1.06			3.40	0.97			3.22	1.06			3.41	0.98			3.17	1.10			3.25	1.12		
Level of education^a^																										
Junior high school	26	3.5	2.85	1.08	0.47^b^	0.757 (0.003)	3.04	0.96	2.08^b^	0.087 (0.012)	3.00	1.23	0.41^b^	0.799 (0.002)	3.73	1.00	3.00^b^	0.021 (0.017)	3.00	1.10	0.75^b^	0.563 (0.004)	2.92	1.29	1.64^b^	0.167 (0.010)
High school	204	27.3	3.10	1.00			3.41	0.90			3.11	1.05			3.32	1.01			3.02	1.15			3.22	1.17		
Technical school, technical college or junior college	127	17.0	3.03	1.04			3.51	0.95			3.23	1.06			3.34	0.95			3.09	1.14			3.36	1.07		
College	333	44.5	3.11	1.07			3.53	0.92			3.19	1.06			3.51	1.00			3.16	1.11			3.40	1.07		
Graduate School	58	7.8	3.05	1.26			3.59	1.01			3.14	1.18			3.12	1.11			3.26	1.18			3.45	1.20		
Occupation*																										
Health care provider	13	1.7	2.69	1.25	−1.33	0.183 (0.373)	4.00	0.71	2.66	0.020 (0.571)	3.31	0.95	0.49	0.621 (0.138)	3.08	1.26	−1.19	0.236 (0.332)	3.54	0.88	1.37	0.170 (0.385)	3.23	1.01	−0.33	0.743 (0.092)
Non‐health care provider	735	98.3	3.09	1.06			3.47	0.93			3.16	1.08			3.41	1.00			3.10	1.13			3.33	1.12		
Family history of cardiovascular diseases*																										
Yes	307	41.0	3.16	1.07	1.68	0.094 (0.125)	3.60	0.91	2.97	0.003 (0.221)	3.40	1.08	5.10	<0.001 (0.379)	3.41	1.02	0.19	0.846 (0.014)	3.39	1.08	5.68	<0.001 (0.423)	3.58	1.04	5.08	<0.001 (0.378)
No or unknown	441	59.0	3.03	1.05			3.39	0.94			3.00	1.04			3.40	1.00			2.92	1.12			3.16	1.14		

*Note*: Unpaired *t*‐test.

Abbreviation: SD, standard deviation.

* Unpaired t‐test.

^a^ One‐way analysis of variance (ANOVA); Welch's ANOVA was used when the homogeneity of variance assumption was violated.

^b^ *F* value (Welch's *F* is reported for Welch's ANOVA). Effect size: Cohen's *d* for *t*‐tests; *η* ^2^ for one‐way ANOVA.

### 3.2 Genomic knowledge (GK) and health numeracy (HN)

The mean GK score was 4.67 (SD = 2.95; possible range 0–10) and the mean HN score was 3.95 (SD = 1.72; range 0–6). Participants with a family history of CVD demonstrated significantly higher GK (mean 5.22) than those without (mean 4.29; *t*(746) = 4.33, *p* < 0.001; Cohen's *d* = 0.32) (Table 1). Healthcare providers scored higher on GK than non‐healthcare providers (mean 7.92 vs. 4.62; *t* = 5.21, *p* < 0.001; Cohen's *d* = 1.13), although the number of healthcare providers was small (*n* = 13). Educational attainment was associated with GK (Welch's *F* = 5.37, *p* < 0.001; *η* ^2^ = 0.028), indicating a small effect size (Table 1). No significant sex differences were observed in GK or HN scores.

The correct answer rates for each GK item are presented in Table 3. The correct answer rates were higher among participants with a family history of CVD for several items (Table 3). Participants with higher educational attainment and healthcare‐related occupations tended to have higher GK scores. HN scores were also higher among married individuals (*p* < 0.001), those with children (*p* < 0.001), and participants with a family history of CVD (*p* = 0.004).

**TABLE 3:** Correct answer rates for genomic knowledge items overall and by family history of cardiovascular disease (*N* = 748).

Item	Genomic knowledge item (statement)	Correct response	Total correct		Family history of CVD (*n* = 307) correct		No/unknown (*n* = 441) correct		*p* value (*χ* ^2^)
			*n*	%	*n*	%	*n*	%	
Q1.	Genetic information does not change over a lifetime	TRUE	293	39.2	137	44.6	156	35.4	0.011
Q2.	Knowledge of genetic information can sometimes predict future disease onset	TRUE	527	70.5	235	76.5	292	66.2	0.002
Q3.	Every child of a person at genetic risk for a disease is at genetic risk for that disease	FALSE	191	25.5	81	26.4	110	24.9	0.657
Q4.	Blood relatives (parents, siblings, children) of a person with a certain genetic variant may have the same genetic variant	TRUE	453	60.6	200	65.1	253	57.4	0.032
Q5.	People who carry a genetic mutation that puts them at risk for a disease will always get that disease	FALSE	369	49.3	158	51.5	211	47.8	0.330
Q6.	Gene mutations are inherited from parents and do not arise anew	FALSE	311	41.6	139	45.3	172	39.0	0.087
Q7.	Cardiovascular diseases such as dilated cardiomyopathy can be inherited	TRUE	233	31.1	126	41.0	107	24.3	<0.001
Q8.	Genetic mutations can change the course of treatment for cardiovascular diseases such as dilated cardiomyopathy	TRUE	257	34.4	125	40.7	132	29.9	0.002
Q9.	In the future, information may be revealed that will change the interpretation of genetic test results	TRUE	430	57.5	200	65.1	230	52.2	<0.001
Q10.	The results of genetic analysis may cause psychological stress	TRUE	432	57.8	202	65.8	230	52.2	<0.001

*Note*: “Incorrect/don't know” includes incorrect and “don't know” responses. *p*‐values were calculated using two‐sided *χ* ^2^ tests comparing the proportion of correct responses between participants with vs. no/unknown family history of CVD.

### 3.3 Perceptions of genetic testing (PGT)

The mean scores for perceived usefulness were 3.08 (SD = 1.06) for item A, 3.48 (SD = 0.93) for item B, and 3.16 (SD = 1.07) for item C. The mean score for perceived fear was 3.41 (SD = 1.01). The mean scores for testing intention (Intention A) and sharing intention (Intention B) were 3.11 (SD = 1.13) and 3.33 (SD = 1.12), respectively (Table 2). Table 2 summarizes the overall scores (top row) and compares the PGT and behavioral intentions according to participant attributes. No significant sex differences were observed in terms of perceived usefulness, fear, or behavioral intentions. Perceived usefulness (particularly items B and C) was significantly higher among participants with a family history of CVD (*p* = 0.003 and *p* < 0.001, respectively), whereas fear did not differ according to family history (*p* = 0.846).

Age group differences were observed for perceived usefulness item A (*p* = 0.012) and item C (*p* < 0.001), and for testing intention (Intention A) (*p* = 0.002), with generally lower scores among participants aged ≥70 years. Educational attainment was associated with perceived fear (*p* = 0.021), whereas perceived usefulness item B was higher among healthcare than non‐healthcare providers (*p* = 0.020). For behavioral intentions, participants with a family history of CVD showed significantly higher scores for both Intention A (*p* < 0.001) and Intention B (*p* < 0.001).

### 3.4 Behavioral intentions toward genetic testing

The mean score for testing intention was 3.11 (SD = 1.13), and that for sharing intention was 3.33 (SD = 1.12). Participants with a family history of CVD exhibited stronger intentions for both testing (*p* < 0.001, Cohen's *d* = 0.42) and sharing (*p* < 0.001, Cohen's *d* = 0.38) than those without. Testing intention was higher in younger age groups (Table 2).

### 3.5 Correlation analysis

Pearson's correlations among the study variables are presented in Table 4. GK was positively correlated with HN (*r* = 0.375, *p* < 0.01), perceived usefulness items (*r* = 0.131–0.354, *p* < 0.01), and intentions (Intention A: *r* = 0.200, Intention B: *r* = 0.288, both *p* < 0.01). HN showed small positive correlations with usefulness item B (*r* = 0.156, *p* < 0.01) and Intention B (*r* = 0.142, *p* < 0.01). Usefulness items were moderately correlated with both intentions (*r* = 0.323–0.486, *p* < 0.01). Fear showed a small negative correlation with Intention A (*r* = −0.103, *p* < 0.01) and was not correlated with Intention B (*r* = −0.036).

**TABLE 4:** Analysis of correlation among variables (*N* = 748).

	Health numeracy score	Perceived usefulness A	Perceived usefulness B	Perceived usefulness C	Perceived fear	Intention A	Intention B
Genomic knowledge score	0.375*	0.131*	0.354*	0.211*	−0.014	0.200*	0.288*
Health numeracy score		−0.044	0.156*	0.015	−0.025	0.006	0.142*
Perceived usefulness A			0.474*	0.320*	0.085**	0.410*	0.323*
Perceived usefulness B				0.354*	−0.014	0.486*	0.473*
Perceived usefulness C					0.097*	0.402*	0.341*
Perceived fear						−0.103*	−0.036
Intention A							0.653*

*Note*: Values are Pearson's correlation coefficients (two‐tailed). *p < 0.01, **p < 0.05.

### 3.6 Factors associated with behavioral intentions: Multiple regression analysis

Multiple regression analyses identified factors associated with the two behavioral intentions (Table 5). For Intention A, the model explained 37.0% of the variance (*R* ^2^ = 0.370; adjusted *R* ^2^ = 0.359). Younger age group (β = −0.090, *p* = 0.008), family history of CVD (β = 0.126, *p* < 0.001), higher perceived usefulness items A–C (β = 0.194–0.294, all *p* < 0.001), and lower fear (β = −0.147, *p* < 0.001) were associated with higher intention to undergo testing.

**TABLE 5:** Multiple regression analysis with behavioral intention as dependent variable (*N* = 748).

	β	*p*	95% CI
Intention A			
Genetic/genomic knowledge score	0.022	0.524	[−0.017, 0.034]
Health numeracy score	−0.026	0.436	[−0.061, 0.026]
Sex	0.003	0.912	[−0.134, 0.150]
Age group	−0.090	0.008	[−0.111, −0.017]
Marital status	−0.048	0.198	[−0.293, 0.061]
Children	0.004	0.921	[−0.167, 0.185]
Level of education	0.024	0.435	[−0.039, 0.091]
Healthcare‐related occupation	0.007	0.809	[−0.449, 0.575]
Family history of cardiovascular diseases	0.126	<0.001	[0.153, 0.426]
Perceived usefulness A	0.194	<0.001	[0.133, 0.278]
Perceived usefulness B	0.294	<0.001	[0.269, 0.443]
Perceived usefulness C	0.208	<0.001	[0.151, 0.287]
Perceived fear	−0.147	<0.001	[−0.230, −0.099]
*R* ^2^	0.370	<0.001	
Adjusted *R* ^2^	0.359		
Intention B			
Genetic/genomic knowledge score	0.109	0.002	[0.015, 0.068]
Health numeracy score	0.068	0.055	[−0.001, 0.089]
Sex	0.017	0.593	[−0.107, 0.187]
Age group	−0.104	0.003	[−0.122, −0.024]
Marital status	0.062	0.111	[−0.034, 0.333]
Children	0.016	0.684	[−0.145, 0.221]
Level of education	0.030	0.359	[−0.036, 0.099]
Healthcare‐related occupation	−0.078	0.014	[−1.200, −0.136]
Family history of cardiovascular diseases	0.100	0.002	[0.085, 0.369]
Perceived usefulness A	0.100	0.006	[0.030, 0.180]
Perceived usefulness B	0.307	<0.001	[0.278, 0.458]
Perceived usefulness C	0.152	<0.001	[0.088, 0.229]
Perceived fear	−0.069	0.027	[−0.145, −0.009]
*R* ^2^	0.308	<0.001	
Adjusted *R* ^2^	0.296		

*Note*: β indicates standardized regression coefficients. 95% CI indicates the confidence interval for the unstandardized coefficient (B). Sex, marital status, children, healthcare‐related occupation, and family history of cardiovascular diseases were entered as binary variables, and age group and level of education were treated as ordinal variables (higher values indicate older age/higher education). All models were estimated using forced‐entry multiple linear regression (ENTER method). *p*‐values are two‐sided.

Abbreviation: CI, confidence interval.

**FIGURE 1:**
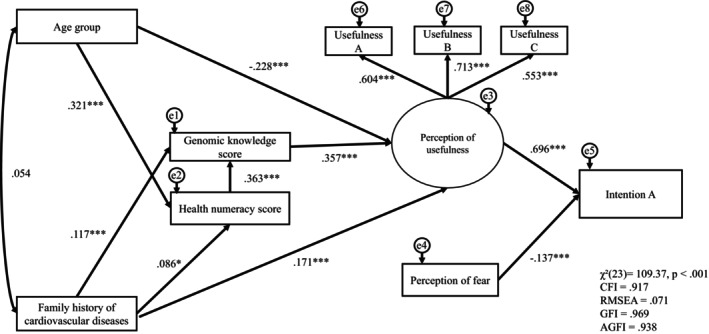
(Intention A): SEM path model of the intention to undergo genetic testing for cardiovascular disease (Intention A) (*N* = 748). Rectangles represent observed variables, and the ellipse represents the latent construct (perceived usefulness), indicated by three observed items (Usefulness A–C). Curved arrows indicate the correlations between the exogenous variables. Values shown are the standardized path coefficients. e1–e8 represent residual (error) terms. Model fit indices were: *χ* ^2^(23) = 109.37, *p* < 0.001; CFI = 0.917; RMSEA = 0.071; GFI = 0.969; AGFI = 0.938. AGFI, adjusted goodness‐of‐fit index; CFI, comparative fit index; GFI, goodness‐of‐fit index; RMSEA, root mean square error of approximation. *p < 0.05, **p < 0.01, ***p < 0.001.

**FIGURE 2:**
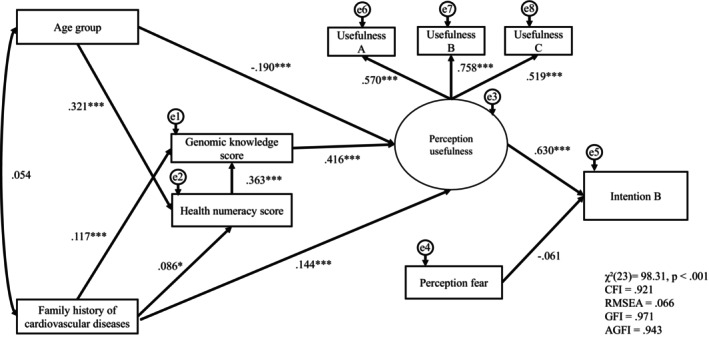
(Intention B): SEM path model of the intention to share genetic testing results with family members (Intention B) (*N* = 748). Rectangles represent observed variables and the ellipse represents the latent construct (perceived usefulness), indicated by three observed items (Usefulness A–C). Curved arrows indicate the correlations between the exogenous variables. Values shown are the standardized path coefficients. e1–e8 represent residual (error) terms. Model fit indices were: *χ* ^2^(23) = 98.31, *p* < 0.001; CFI = 0.921; RMSEA = 0.066; GFI = 0.971; AGFI = 0.943. AGFI, adjusted goodness‐of‐fit index; CFI, comparative fit index; GFI, goodness‐of‐fit index; RMSEA, root mean square error of approximation. *p < 0.05, **p < 0.01, ***p < 0.001. Asterisks indicate ML‐based *p* values; for the fear → Intention B path, the ML *p* value was 0.047, but the bias‐corrected bootstrap standardized 95% CI (5000 resamples) included zero; therefore, no asterisk is shown for that path.

For Intention B, the model explained 30.8% of the variance (*R* ^2^ = 0.308; adjusted *R* ^2^ = 0.296). GK (β = 0.109, *p* = 0.002), younger age group (β = −0.104, *p* = 0.003), family history of CVD (β = 0.100, *p* = 0.002), higher perceived usefulness items A–C (β = 0.100–0.307; *p* = 0.006 to <0.001), and lower fear (β = −0.069, *p* = 0.027) were weakly associated with higher sharing intention, whereas healthcare‐related occupations were significantly associated with lower sharing intention (β = −0.078, *p* = 0.014). No multicollinearity was detected (VIF range, 1.11–1.68). Among the factors included in the models, the perceived usefulness items showed the largest standardized coefficients in both models. Note that usefulness was entered as three separate items (A–C) in the main regressions (Table 5), whereas the summed usefulness score was used in the sensitivity analyses (Table S2), and a latent usefulness construct was used in the SEM path model (Figures 1 and 2).

### 3.7 Sensitivity analyses stratified by cardiovascular condition group

The sensitivity analyses stratified by cardiovascular condition group, including a proxy defined predominant monogenic component subgroup and an acquired/multifactorial only subgroup, are shown in Table S2. In both strata, perceived usefulness showed the largest coefficients for both intentions, and perceived fear was negatively associated with testing intention. Coefficient magnitudes differed for some paths across strata (e.g., HN and perceived usefulness).

### 3.8 SEM path model: Structural model of behavioral intention

We tested two SEM path models corresponding to Intention A (intention to undergo genetic testing) and Intention B (intention to share test results with the family). In both models, age group and family history of CVD were specified as exogenous variables and allowed to correlate (*r* = 0.054). Perceived usefulness was modeled as a latent construct indicated by three observed items (Usefulness A–C), whereas perceived fear was modeled as a single‐item indicator. The standardized path coefficients are presented in Figures 1 and 2.

Intention A model (Figure 1). Model fit was acceptable: *χ* ^2^(23) = 109.37, *p* < 0.001; CFI = 0.917; RMSEA = 0.071; GFI = 0.969; AGFI = 0.938. Age group was positively associated with HN (β = 0.321, *p* < 0.001) and negatively associated with perceived usefulness (β = −0.228, *p* < 0.001). A family history of CVD was positively associated with GK (β = 0.117, *p* < 0.001), HN (β = 0.086, *p* < 0.05), and perceived usefulness (β = 0.171, *p* < 0.001). HN was positively associated with GK (β = 0.363, *p* < 0.001), and GK was positively associated with perceived usefulness (β = 0.357, *p* < 0.001). Perceived usefulness showed a strong positive association with Intention A (β = 0.696, *p* < 0.001), whereas perceived fear showed a negative association (β = −0.137, *p* < 0.001). Factor loadings for the perceived usefulness construct were significant (Usefulness A: λ = 0.604, *p* < 0.001; Usefulness B: λ = 0.713, *p* < 0.001; Usefulness C: λ = 0.553, *p* < 0.001). The model explained 50.4% of the variance in Intention A (*R* ^2^ = 0.504).

Intention B model (Figure 2). Model fit was acceptable: *χ* ^2^(23) = 98.31, *p* < 0.001; CFI = 0.921; RMSEA = 0.066; GFI = 0.971; AGFI = 0.943. Age group and family history showed the same upstream pattern as in the Intention A model (age group → HN: β = 0.321, *p* < 0.001; family history → HN: β = 0.086, *p* < 0.05; family history → GK: β = 0.117, *p* < 0.001; HN → GK: β = 0.363, *p* < 0.001). GK was positively associated with perceived usefulness (β = 0.416, *p* < 0.001), while age group was negatively associated with perceived usefulness (β = −0.190, *p* < 0.001), and family history was positively associated with perceived usefulness (β = 0.144, *p* < 0.001). Perceived usefulness was positively associated with Intention B (β = 0.630, *p* < 0.001), whereas the direct path from fear to Intention B was small (β = −0.061) and not robust: the ML‐based *p*‐value was 0.047, but the bias‐corrected bootstrap standardized 95% CI (5000 resamples) included zero; therefore, no asterisk is shown for that path in Figure 2. Loadings for perceived usefulness were significant (Usefulness A: λ = 0.570, *p* < 0.001; Usefulness B: λ = 0.758, *p* < 0.001; Usefulness C: λ = 0.519, *p* < 0.001). The model explained 40.1% of the variance in Intention B (*R* ^2^ = 0.401). Indirect effects were evaluated using bias‐corrected bootstrapping (5000 resamples).

## 4 DISCUSSION

This study examined the associations between GK, HN, and PGT with the behavioral intention to undergo and share genetic testing for CVD among Japanese individuals with self‐reported physician‐diagnosed CVD. The results were broadly consistent with the proposed conceptual model: cognitive factors (GK and HN) were associated with behavioral intentions primarily through perceptions of the usefulness of genetic testing. Family history of CVD was associated with higher knowledge and perceived usefulness and, through these pathways, with stronger behavioral intentions, whereas fear was negatively associated with testing intention (Ferrer & Klein, 2015; Janz & Becker, 1984; Rogers, 1975). Overall, the findings clarify the psychological mechanisms underlying testing decisions in CVD and highlight areas for improvement in genetic counseling and patient education (Oliveri et al., 2018; Sweeny et al., 2014).

### 4.1 Genomic literacy and the role of knowledge

Consistent with previous research on genomic literacy, participants demonstrated a partial understanding of basic genetic concepts, with relatively high comprehension of predictability but lower understanding of the immutability and shareability of genetic information (Miyoshi & Watanabe, 2021a, 2023). This pattern is broadly consistent with prior discussions that genetic information can be misunderstood in the public sphere and may raise distinctive concerns regarding privacy, stigma, and familial implications (Barlow‐Stewart et al., 2009; Green & Botkin, 2003). The overall level of GK observed in this study was modest. This pattern is broadly consistent with prior findings, suggesting that limited genomic literacy is not uncommon in Japan.

Participants with a family history of CVD exhibited higher GK scores. This pattern suggests that family history may be linked to greater awareness of and engagement with genetic information. Family history may function as a cognitive and emotional cue to action, potentially reflecting greater perceived personal relevance and information seeking, consistent with mechanisms described in the HBM (Janz & Becker, 1984; Rosenstock, 1974). Importantly, GK was not directly associated with the intention to undergo testing but was significantly linked to the perception of its usefulness. This supports the idea that knowledge alone is insufficient for behavior change, echoing findings from genetic testing decision‐making research (Oliveri et al., 2018; Sweeny et al., 2014). Individuals may possess a factual understanding but remain ambivalent or fearful about applying that knowledge to personal decisions. In this context, knowledge serves as a foundation, but perceived relevance and confidence mediate its translation into practice.

### 4.2 Health numeracy as foundational competence

HN had a significant indirect effect on behavioral intention via GK and perceived usefulness. This indicates that numerical ability may facilitate the comprehension of quantitative and probabilistic genetic information, which, in turn, enhances perceived benefits. Previous studies have shown that numeracy contributes to interpreting risk probabilities, assessing treatment options, and evaluating uncertainties (Cokely & Kelley, 2009; Garcia‐Retamero et al., 2019; Peters, 2008).

Our findings extend these results to the genomic domain by suggesting that patients with higher numeracy may better understand key probabilistic concepts relevant to cardiogenetic testing—such as a 50% transmission risk in autosomal dominant inheritance, age‐dependent penetrance and residual risk, and uncertainty in variant interpretation (e.g., variants of uncertain significance)—and how these may inform surveillance and family risk management (Peters, 2008; Wilde et al., 2022). However, HN did not show a robust direct association with behavioral intentions in the main multivariable models after accounting for GK and perceived usefulness or fear. One possible explanation is that intentions are shaped more proximally by perceived usefulness and affective/ethical considerations than by general numeracy skills (Ferrer & Klein, 2015; Sweeny et al., 2014).

In addition, prior disease‐specific work in hypertrophic cardiomyopathy has shown that a better comprehension of autosomal dominant inheritance predicts the communication of genetic risk information to relatives (Batte et al., 2015). This discrepancy may reflect differences in measurement (inheritance‐specific comprehension vs. general HN), outcomes (actual family communication vs. self‐reported intentions), and sample composition (HCM‐focused at‐risk samples vs. a broader CVD population, including multifactorial conditions). This pattern is consistent with deliberative and affective pathways in health decision‐making, in which affective responses can sometimes override analytical processing when decisions involve perceived threats or identity implications (Ferrer & Klein, 2015). Hence, educational interventions that combine cognitive and emotional components—rather than focusing solely on factual numeracy—may be more effective in improving testing uptake (Oliveri et al., 2018; Sweeny et al., 2014).

### 4.3 Perceptions of genetic testing: Usefulness and fear

Among all variables, perceived usefulness was the factor most strongly associated with the intentions to undergo genetic testing and share results with family members. This highlights the central role of perceived benefits in motivating engagement with genomic medicine (Janz & Becker, 1984; Rogers, 1975; Sweeny et al., 2014). Patients who viewed genetic testing as helpful for “planning their future” or as providing potentially meaningful health‐related information were more likely to express willingness to undergo testing, consistent with prior work indicating that perceived benefits are major drivers of genetic testing decisions (Oliveri et al., 2018; Sweeny et al., 2014).

Fear of genetic testing showed a modest negative association with the intention to undergo testing, consistent with affective barriers such as anxiety about unfavorable results (Helmes, 2002; Oliveri et al., 2018). In contrast, its association with the intention to share results with family members was very small and not robust in bootstrapping (ML *p* = 0.047, but the bias‐corrected bootstrap standardized CI included zero), suggesting that fear may be more relevant to one's personal testing decision than to the intention to communicate with family members. This fear may stem from concerns about unfavorable results, potential discrimination, or emotional distress for oneself or family members (Barlow‐Stewart et al., 2009; Green & Botkin, 2003; Oliveri et al., 2018). Although the magnitude of this association was modest, it underscores the need for emotionally supportive counseling that addresses patients' anxieties, normalizes uncertainty, and promotes informed—not coerced—decisions (Sweeny et al., 2014). Notably, perceived fear did not fully negate the influence of usefulness, suggesting that perceived benefits may mitigate emotional barriers when they are adequately communicated. Thus, improving the clarity and accessibility of benefit‐related information is essential for countering fear‐based avoidance.

### 4.4 Family history and the intergenerational nature of CVD

The inclusion of family history as an antecedent variable was one of the distinguishing features of this study. Family history of CVD was associated with higher GK and stronger intentions to both undergo testing and share results with family members. These findings are consistent with previous studies suggesting that perceived familial disease risk is related to higher risk perception and greater salience of family‐related considerations in health decision‐making (Davison et al., 1992; Hunt et al., 2000).

In our SEM path model, family history was positively associated with GK and perceived usefulness (and weakly with HN), which is consistent with the possibility that familial experience may function as a cue to action and may be linked to greater engagement with genetic information and stronger benefit‐related perceptions (Hunt et al., 2000; Janz & Becker, 1984). Because cardiogenetic test results can have implications for multiple relatives, greater awareness of hereditary risk may be associated with a higher intention to communicate results within families (Ho et al., 2022; Wilde et al., 2022). Simultaneously, family‐related considerations can introduce emotional and ethical tensions regarding disclosure (e.g., worry about relatives, guilt, stigma, or uncertainty about whether and how to share results), underscoring the importance of structured, family‐centered counseling and communication support (Barlow‐Stewart et al., 2009; Green & Botkin, 2003). Given the cross‐sectional design, these associations should be interpreted as correlational. Nevertheless, our findings highlight the potential value of counseling approaches that address both the perceived usefulness and practical needs of family communication to support informed decision‐making (Burns et al., 2023; Ho et al., 2022).

### 4.5 Integrating findings with existing models

These findings align with the HBM and PMT, which state that health‐related actions are shaped by both threat appraisal (e.g., fear) and coping appraisal (e.g., perceived usefulness) (Janz & Becker, 1984; Rogers, 1975; Rosenstock, 1974). Consistent with these frameworks, our path model indicated that cognitive factors (GK and HN) were associated with behavioral intentions primarily through perceived usefulness (Ferrer & Klein, 2015; Sweeny et al., 2014). Importantly, cardiogenetics research suggests that improving knowledge alone may be insufficient to increase familial communication of genetic results; a randomized controlled trial in hypertrophic cardiomyopathy reported higher genetic knowledge without a statistically significant improvement in the primary family communication outcome (Burns et al., 2023). Together, these findings support interventions that address both benefit appraisal and emotional/communication barriers. In this framing, perceived usefulness reflects coping appraisal, whereas fear reflects threat appraisal, helping explain why usefulness showed robust associations with both intentions, whereas fear was more specific to personal testing intention.

### 4.6 Comparison with previous research

While previous research on genomic literacy among the general public has identified low baseline knowledge, our nationwide web‐based sample of individuals with self‐reported physician‐diagnosed cardiovascular conditions—many of which are multifactorial or acquired—suggests that knowledge gaps persist among people living with CVD in the general population (Miyoshi & Watanabe, 2021b, 2023). For example, in a nationwide web‐based survey of Japanese adults, the mean objective GK score was approximately 46% correct (8.31 out of 18 items) (Miyoshi & Watanabe, 2021b), although direct numerical comparison is limited because of differences in item composition and context. Moreover, the strong mediating role of perceived usefulness indicates that interventions focusing solely on knowledge dissemination may have a limited impact unless they also address perceived personal relevance and emotional readiness (Ferrer & Klein, 2015; Sweeny et al., 2014).

In clinical practice, this misunderstanding could lead to unrealistic expectations regarding lifestyle modifications or overconfidence in the reversibility of genetic predisposition. Thus, improving the comprehension of both genetic determinism and modifiability is crucial for realistic risk communication (Green & Botkin, 2003; Peters, 2008).

### 4.7 Limitations

This study has some limitations. First, the cross‐sectional design precludes causal inference; the observed associations among genomic literacy, perceptions, and intentions should be interpreted as correlational. Moreover, our outcomes captured self‐reported intentions rather than the actual uptake of genetic testing or result sharing, and an intention–behavior gap may limit clinical interpretation. Second, participants were recruited from an online research panel; although demographic quotas were applied, selection bias is possible, and generalizability to clinic‐based patient populations may be limited (e.g., differences in disease severity, access to specialty care, and health/digital literacy). Third, CVD diagnoses and family history were self‐reported and could not be verified using medical records, raising the possibility of recall bias or misclassification. Fourth, our sample included individuals with a broad range of cardiovascular conditions, many of which are multifactorial or acquired rather than classical indications for cardiogenetic panel testing. Although we adopted this inclusive approach to capture diverse cardiovascular experiences, participants may have interpreted “genetic testing for CVD” heterogeneously. In addition, because no standardized definition or explanatory description of cardiogenetic testing was provided prior to the relevant survey items and participants responded based on the item stems only, this heterogeneity in interpretation may have introduced measurement error in intention‐related responses. Fifth, limitations related to the GK scale should be acknowledged. Although the 10‐item GK scale was designed to assess general genomic concepts and CVD‐related testing knowledge, two items (Q7 and Q8) specifically referred to dilated cardiomyopathy. Because our sample comprised individuals with a broad range of self‐reported physician‐diagnosed cardiovascular conditions, these disease‐specific items may not have been equally applicable or easily interpretable by all participants. Respondents without familiarity with inherited cardiomyopathies may have answered these items based on limited exposure, potentially leading to an underestimation of GK for some subgroups and reduced content validity for the overall CVD population. Such measurement non‐equivalence would be expected to attenuate associations rather than inflate them; nonetheless, the interpretation of absolute GK levels should be made with caution, and future studies should refine and validate cardiogenetics knowledge measures that are diagnosis‐agnostic or tailored to specific clinical contexts. Sixth, an additional measurement issue should be noted for the perceived usefulness construct. One usefulness item (“Genetic testing can reduce the risk of any disease”) may have captured a generalized positive belief or misconception about genetic testing rather than perceived clinical utility per se. Accordingly, the usefulness construct in this study should be interpreted as reflecting participants' positively valenced perceptions of testing, rather than a purely accurate appraisal of medical utility. Finally, perceived fear was assessed using a single item and therefore served only as a brief indicator. It cannot distinguish fear from related constructs, such as anxiety or worry, and fear‐related findings should be interpreted cautiously. Despite these limitations, this study leveraged a nationwide sample and theory‐informed modeling to examine how GK, HN, and PGT are jointly related to genetic testing intentions among individuals with CVD.

### 4.8 Practical and educational implications

These findings have direct implications for the design of patient education and genetic counseling strategies. First, enhancing genomic literacy—particularly understanding genetic immutability and familial implications—may support readiness for testing (Green & Botkin, 2003; Miyoshi & Watanabe, 2021a). Second, numeracy‐based educational tools (e.g., risk probability visuals) may help patients interpret the results and reduce misperceptions (Peters, 2008). Third, interventions should integrate affective components, such as empathy‐focused counseling, to address fear and anxiety (Helmes, 2002; Oliveri et al., 2018). Fourth, structured communication support for family result sharing could help patients navigate disclosure while minimizing feelings of guilt or stigma (Burns et al., 2023; Ho et al., 2022).

For healthcare systems, these results underscore the need for interdisciplinary education bridging genomic medicine, psychology and communication science (Ferrer & Klein, 2015; Sweeny et al., 2014). Incorporating these elements into cardiogenetic counseling protocols may foster informed decision‐making and promote the ethical implementation of precision medicine in Japan (Arbelo et al., 2023; Wilde et al., 2022). Importantly, the availability and scope of practice of genetic counselors vary substantially across countries and healthcare systems; in many settings (including Japan), cardiogenetic counseling and return of results may be delivered by clinical geneticists, cardiologists, nurses, or multidisciplinary teams rather than by independent genetic counselors. Accordingly, these interdisciplinary competencies should be incorporated into training and care pathways for all relevant professionals, supported by scalable approaches, such as structured counseling protocols, decision aids, and team‐based workflows.

From a genetic counseling perspective, strategies that explicitly target perceived usefulness, GK, and numeracy may support more informed decision‐making among patients. Healthcare professionals should incorporate educational interventions that address both cognitive and perceptual gaps when communicating about genetic testing for CVD. Given the cross‐sectional design of this study, these implications should be interpreted as hypothesis‐generating, rather than causal. However, they provide a concrete basis for developing and evaluating future educational and counseling programs.

## 5 CONCLUSION

This study found that GK and HN were indirectly associated with behavioral intentions toward CVD genetic testing through perceived usefulness. Family history of CVD was associated with both knowledge and intentions, whereas fear showed a modest negative association. These findings suggest that enhancing genomic literacy alone may be insufficient, and educational efforts must also address the emotional and perceptual factors that mediate decision‐making. For genetic counselors and healthcare professionals, tailoring communication to patients' cognitive and emotional profiles may strengthen informed consent and engagement in genetic testing. Future research should employ longitudinal or intervention designs to confirm causal pathways and evaluate educational programs that integrate both cognitive and affective dimensions of genomic literacy.

## AUTHOR CONTRIBUTIONS

Conceptualization: Tomomi Miyoshi, Atsushi Mizuno, Seitaro Nomura. Data curation: Atsushi Mizuno, Koichiro Isa. Formal analysis: Tomomi Miyoshi, Masaki Watanabe. Funding acquisition: Seitaro Nomura. Investigation: Atsushi Mizuno, Koichiro Isa. Methodology: Tomomi Miyoshi, Atsushi Mizuno. Project administration: Seitaro Nomura. Resources: Atsushi Mizuno, Seitaro Nomura. Software: Tomomi Miyoshi, Masaki Watanabe. Supervision: Atsushi Mizuno, Seitaro Nomura. Validation: Tomomi Miyoshi, Masaki Watanabe. Visualization: Tomomi Miyoshi. Writing – original draft: Tomomi Miyoshi. Writing – review and editing: Atsushi Mizuno, Seitaro Nomura, Koichiro Isa, Masaki Watanabe, Toru Kubo, Kanna Fujita.

## FUNDING INFORMATION

This work was supported by grants from the Japan Agency for Medical Research and Development (AMED) (JP21ek0109543, JP18km0405209, JP24ek0109755, JP23tm0524009, and JP23tm0524004).

## CONFLICT OF INTEREST STATEMENT

None declared.

## ETHICS STATEMENT

This study was approved by the Research Ethics Committee of the Faculty of Medicine, The University of Tokyo (approval no. 2022166G).

Human studies and informed consent: Electronic informed consent was obtained from all participants prior to participation in the study.

Animal studies: No animals were involved in this study.

## Supporting information


Tables S1–S3

## Data Availability

The data supporting the findings of this study are available from the corresponding author upon reasonable request. Owing to privacy regulations and contractual agreements with the web research company, raw individual‐level data cannot be publicly shared.
